# Bioconvective Reiner–Rivlin nanofluid flow over a rotating disk with Cattaneo–Christov flow heat flux and entropy generation analysis

**DOI:** 10.1038/s41598-021-95448-y

**Published:** 2021-08-04

**Authors:** Yu-Pei Lv, Hina Gul, Muhammad Ramzan, Jae Dong Chung, Muhammad Bilal

**Affiliations:** 1 Department of Mathematics, Huzhou University, Huzhou, 313000 People’s Republic of China; 2 Department of Computer Science, Bahria University, Islamabad, 44000 Pakistan; 3 Department of Mechanical Engineering, Sejong University, Seoul, 143-747 Korea; 4 University of Lahore, Gujrat Campus, Gujrat, 50700 Pakistan

**Keywords:** Software, Mechanical engineering

## Abstract

The non-Newtonian fluids possess captivating heat transfer applications in comparison to the Newtonian fluids. Here, a new type of non-Newtonian fluid named Reiner–Rivlin nanofluid flow over a rough rotating disk with Cattaneo–Christov (C–C) heat flux is studied in a permeable media. The stability of the nanoparticles is augmented by adding the gyrotactic microorganisms in the nanofluid. The concept of the envisaged model is improved by considering the influences of Arrhenius activation energy, chemical reaction, slip, and convective conditions at the boundary of the surface. The entropy generation is evaluated by employing the second law of thermodynamics. The succor of the Shooting scheme combined with the bvp4c MATLAB software is adapted for the solution of extremely nonlinear system of equations. The noteworthy impacts of the evolving parameters versus engaged fields are inspected through graphical illustrations. The outcomes show that for a strong material parameter of Reiner–Rivlin, temperature, and concentration profiles are enhanced. The behavior of Skin friction coefficients, local Nusselt number, Sherwood number, and local density number of motile microorganisms against the different estimates of emerging parameters are represented in tabular form. The authenticity of the intended model is tested by comparing the presented results in limiting form to an already published paper. A proper correlation between the two results is attained.

## Introduction

Fluids that do not abide by the Newtonian constitutive relations like large molecular weight polymers are commonly used in lubricants, nylon, blood, clay, detergents, and paints, etc. are named as non-Newtonian fluids. The stress within the viscoelastic fluids remained alive up to some extent upon the removal of stress forces owing to the strong binding of intermolecular trussing. This inimitable feature is characterized as the memory effect. Reiner^1^ and Rivlin^2^ introduced a new kind of non-Newtonian fluid that adequately forecast the flow behavior of numerous biological, geological materials together with polymers and various food products. Later, Kosterin^3^ disclosed that Reiner–Rivlin rheological relation is inappropriate in articulating the influences of normal stresses. The flow of Reiner–Rivlin fluid past rectangular ducts is analyzed numerically by Gao and Hartnett^4^. The interesting outcome of this study revealed that the heat transfer increases substantially in attendance of the second normal stresses. Attia^5^ obtained a numerical solution of the unsteady flow of Reiner–Rivlin fluid past a rotating permeable disk with impacts of suction/injection. The major conclusion of this study is that the heat transfer effect is more significant in the presence of suction in comparison to the injection. In another study Attia^6^ discussed the flow of the Reiner–Rivlin liquid with Ion slip and Hall current impacts past a rotating disk. An interesting result of this investigation points out that the effect of the Ion slip on the axial velocity is more obvious for Reiner–Rivlin fluid as compared to any Newtonian liquid. The numerical solution of the Reiner–Rivlin fluid flow with partial slip owing to a rotating disk is deliberated by Tabassum and Mustafa^7^. This research disclosed that higher values of the torque and slip parameter are needed to keep the steady rotation of the disk. The study of^7^ is extended by Naqvi et al.^8^ discussing the numerical solution of the Reiner–Rivlin liquid flow due to a rotating disk with multiple slips. A decline in the radially outward flow is perceived owing to an upsurge in the Reiner–Rivlin liquid parameter is a key outcome of this study. In a recent study, Rashid and Mustafa^9^ discussed the heat transfer with entropy generation analysis of Von Karman flow owing to a rotating disk of the Reiner–Rivlin liquid. The salient result of this study is that in the case of the Reiner–Rivlin fluid the moment coefficient is significantly decreased.

The bacteria and the microalgae possess high density in comparison to the water and because of this fact they move in an upward direction opposite to gravity. Owing to this phenomenon the top layer becomes thicker than the bottom one and creates an unhinged situation as far as density distribution is concerned. Following the physics of the problem, convective patterns are formed because of the convective instability in this case. Such random instantaneous pattern activity of microorganisms is termed as the bioconvection. Bioconvection is categorized as geotactic^10, 11^, gyrotactic^12, 13^, and chemotactic^14^. Bioconvection applications may be found in numerous industrial, ecological, and commercial products including fertilizers, ethanol, ecological fuels, and fuel cells. Waqas et al.^15^ studied numerically the flow of Oldroyd-B nanoliquid with motile microorganism impact past a rotating disk. The numerical solution of the transient flow of the rate type thin film nanofluid flow over a rotating disk with bioconvection, activation energy, and Ohmic heating is studied by Abdelmalek et al.^16^. It is inferred from this study that axial and tangential velocities are reduced when the buoyancy ratio parameter is boosted. Ramzan et al.^17^ uncovered a numerical solution of the nanofluid flow containing carbon nanotubes with dust particles over an inclined rotating disk. It is witnessed in this exploration that the thermal field is stronger in the case of the nanofluid phase in comparison to the dust phase and this effect becomes stronger when the impact of the thermal radiation is augmented. Some recent explorations featuring flow over the rotating disk may be found in^18–21^.

In a system and its surroundings, entropy tests the rate of disorder. It is a physical occurrence of heat transfer in the form of energy. Because of any friction or dislocation of particles, the movement of heat induces a difference in potential energy and kinetic energy etc. Bejan^22^ was a initiator, who studied the entropy generation analysis during heat losing procedure of fluid motion. Ijaz et al.^23^ deliberated the Sisko nanofluid with activation energy and entropy generation in non-linear radiative heat flow. It is noticed that entropy generation is increased for mounting values of the Brinkman number. Wakeel et al.^24^ examined the Hall effects in second-grade fluid flow with C–C heat flux and entropy generation over a rotating stretchable disk. The flow of Marangoni Maxwell fluid past a rotating disk accompanying the thermal radiation and activation energy is examined numerically by Devi and Mabood^25^. The salient outcome highlighting the entropy generation impact is that the higher estimates of the Bejan number and the fluid parameter weaken the entropy generation rate. Abbas et al.^26^ discussed the numerical solution of the MHD nanofluid flow over a rotating disk with second-order velocity slip and activation energy with entropy minimization optimization. The main finding of the envisioned model is that the entropy generation is influenced by the magnetic field. Some latest publications focusing on the impact of entropy may be found in^27–29^.

It is a learned phenomenon that the transfer of heat arises due to temperature differences amongst two different objects or within a similar body. Fourier law (heat conduction) possesses a drawback that any disturbance instigated in the beginning will carry out throughout the process. To resolve this issue, Cattaneo introduced thermal relaxation time for Fourier's law (heat conduction) which allows the transport of heat by waves propagating with controlled speed^30^. Later, Christov developed the relation proposed by Cattaneo through frame-indifferent change with the Oldroyd upper-convected derivative. Such relation is entitled as C–C flux model. Shehzad et al.^31^ studied MHD incompressible Maxwell bioconvection fluid flow over a rotating isolated disk in the presence of C–C heat flux. The flow of Oldroyd-B fluid with C–C heat flux over a rotating disk is studied by utilizing the BVP Midrich numerical technique. It is noticed in this exploration that the fluid velocity is affected by the thermal relaxation parameter. Hayat et al.^32^ using Homotopy Analytic scheme found an analytical solution of the Newtonian fluid over a rotating disk having variable thickness with C–C heat flux. The key observation revealed that the surface drag coefficient is affected when the thickness of the disk is increased. Recent studies focusing on the impact of C–C heat flux may be found in^33–35^.

The aforementioned studies disclosed that abundant researches are available in the literature discussing the flow of fluid over a rotating disk. Nevertheless, restricted literature may be found that deliberates the Reiner–Rivlin nanofluid flow over a rotating disk. But no study so far is not attempted that ponders the flow of Reiner–Rivlin nanofluid over a rotating disk with bioconvection, Arrhenius activation energy, C–C heat flux, and entropy generation analysis. The numerical result to the model is found. The abundant applications related to nanofluids may be found in the engineering and industrial processes including electronics applications, transportation, Nanofluids-based microbial fuel cell, industrial cooling applications, Energy storage, Heating buildings and reducing pollution, Space and defense, Magnetic sealing, Antibacterial activity, Nanodrug delivery, Intensify microreactors, Nuclear systems cooling, Mass transfer enhancement, Solar absorption, Mechanical applications, Friction reduction, Nanofluids as vehicular brake fluids, and nanofluids with unique optical properties. The inimitability of the current model as portrayed in Table 1 by associating the present model with the published studies.

**Table 1:** Literature survey for novelty of the envisioned model.

Authors	Reiner–Rivlin flow model	Nanofluid flow over a rotating disk	Cattaneo–Christov flow heat flux	Impact of Bioconvection	Entropy generation analysis	Arrhenius activation energy, chemical reaction
Tabassum et al.^7^	Yes	No	No	No	No	No
Naqvi et al.^8^	Yes	Yes	No	No	No	No
Rashid et al.^9^	Yes	No	Yes	No	Yes	No
Present	**Yes**	**Yes**	**Yes**	**Yes**	**Yes**	**Yes**

## Mathematical modeling

**Figure 1:**
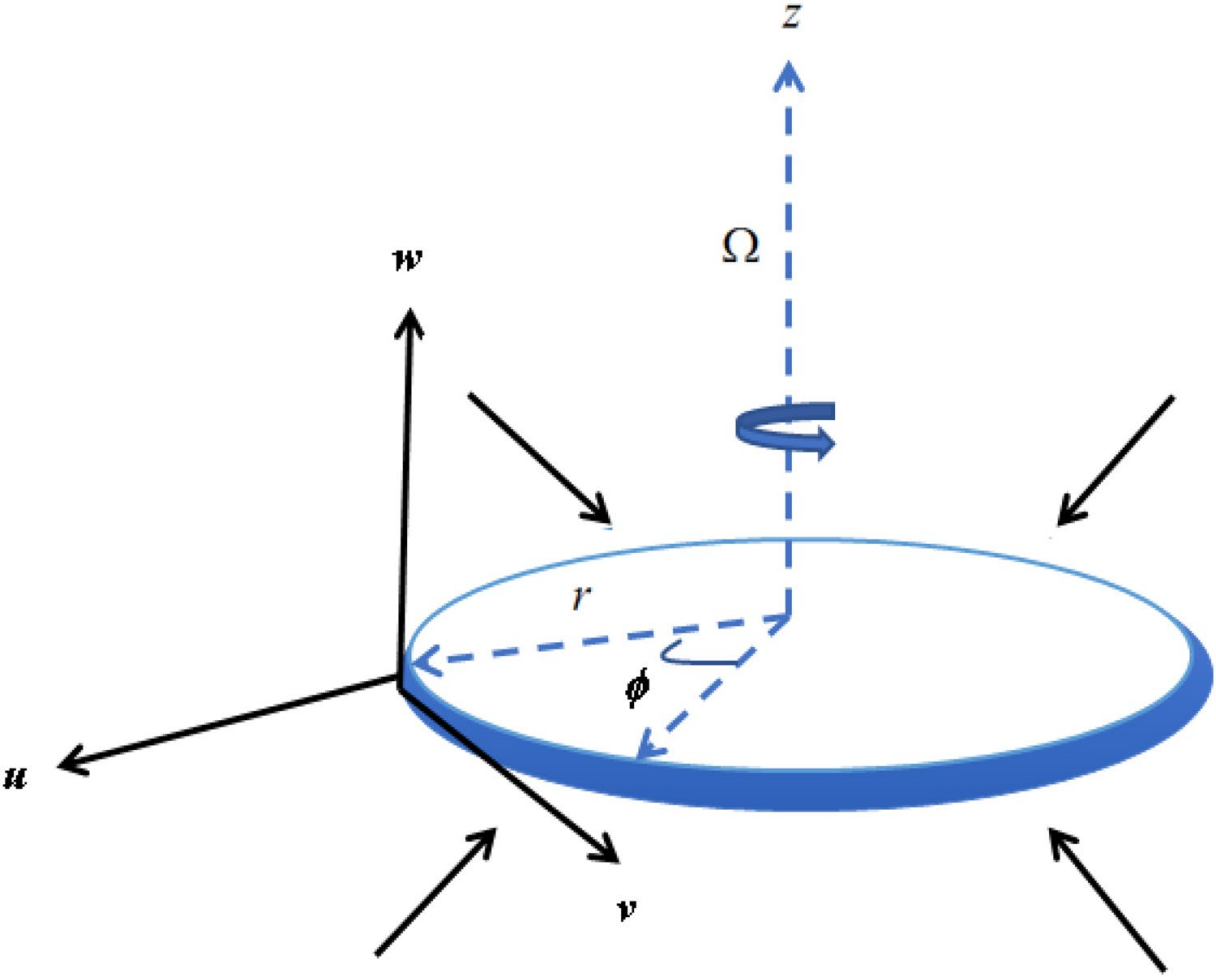
Flow geometry.

Consider a steady axial symmetric three-dimensional incompressible Reiner–Rivlin nanofluid flow rotating with the angular velocity $\omega$ with $z > 0,$ over a rotating disk coinciding with the plane $z = 0,$ (Fig. 1). Let $u,v$ and $w$ be the velocity components along the directions of growing $r,\varphi$ and $z$ respectively. Because of the axial symmetry, the velocity components are assumed to be independent of the azimuthal coordinate $\varphi$^7^. The slip and convective boundary conditions are applied. The impact of activation energy, gyrotactic microorganism, and C–C heat flux in a porous medium are also considered. Entropy generation is also a part of this model.

Following Reiner^1^ and Rivlin^2^, we have the following stress tensor for the flow field:

1 $$\tau_{ij} = - p\delta_{ij} + \mu e_{ij} + \mu_{c} e_{ik} e_{kj} ;e_{jj} = 0.$$

Here $\mu_{c}$ is the coefficient of cross-viscosity, $\delta_{ij}$ is the Kronecker delta, $e_{ij}$ is the tensor of deformation rate and $p$ is the pressure. The governing boundary layer equations under the impact of C–C heat flux are represented as:

2 $$u_{r} + \frac{u}{r} + w_{z} = 0$$

3 $$\rho \left( {uu_{r} + wu_{z} - \frac{{v^{2} }}{r}} \right) = \left( {\tau_{rr} } \right)_{r} + \left( {\tau_{rz} } \right)_{r} + \frac{{\tau_{rr} - \tau_{\varphi \varphi } }}{r} - \frac{\nu }{k}u,$$

4 $$\rho \left( {uv_{r} + wv_{z} + \frac{uv}{r}} \right) = \frac{1}{{r^{2} }}\left( {\left( {2r\tau_{r\varphi } } \right) + r^{2} \left( {\tau_{r\varphi } } \right)_{r} } \right) + \left( {\tau_{z\varphi } } \right)_{z} + \frac{{\tau_{r\varphi } - \tau_{\varphi r} }}{r} - \frac{\nu }{k}v,$$

5 $$\rho \left( {uw_{r} + ww_{z} } \right) = \frac{1}{r}\left( {\tau_{rz} + r(\tau_{rz} )_{r} } \right) + \tau_{zz} - \frac{\nu }{{k^{*} }}w,$$

6 $$\begin{aligned} uT_{r} + wT_{z} & = \alpha_{m} \left( {T_{rr} + \frac{1}{r}T_{r} + T_{zz} } \right) + \frac{{(\rho c)_{p} }}{{(\rho c)_{f} }}\left( {\begin{array}{*{20}l} {D_{B} \left( {T_{r} C_{r} + T_{z} C_{z} } \right)} \hfill \\ { + \frac{{D_{T} }}{{T_{\infty } }}\left( {\left( {T_{r} } \right)^{2} + \left( {T_{z} } \right)^{2} } \right)} \hfill \\ \end{array} } \right) \\ & \quad - \lambda_{2} \left( {u^{2} T_{rr} + w^{2} T_{zz} + 2uwT_{rz} + \left( {uu_{r} + wu_{z} } \right)T_{r} + \left( {uw_{r} + ww_{z} } \right)T_{z} } \right) + q^{*}, \\ \end{aligned}$$

7 $$q^{*} = \frac{{k_{\infty } U_{w} }}{{z\nu (\rho c)_{f} }}\left\{ {A^{*}(T_{w} - T_{\infty } )f + B^{*}(T - T_{\infty } )} \right\}$$

8 $$uC_{r} + wC_{z} = D_{B} \left( {C_{rr} + \frac{1}{r}C_{r} + C_{zz} } \right) + \frac{{D_{T} }}{{T_{\infty } }}\left( {T_{rr} + \frac{1}{r}T_{r} + T_{zz} } \right) - k_{r}^{2} \left( {C - C_{\infty } } \right)\left( {\frac{T}{{T_{\infty } }}} \right)^{n} e^{{\left( {\frac{{ - E_{a} }}{kT}} \right)}} ,$$

9 $$uN_{r} + wN_{z} + \frac{{bW_{c} }}{{C_{w} - C_{\infty } }}\left( {N_{z} C_{z} + NC_{z} } \right) = D_{n} \left( {N_{rr} + \frac{1}{r}N_{r} + N_{zz} } \right).$$

The associated boundary conditions are given as:

10 $$\begin{aligned} u(r,0) & = \beta_{1} \tau_{rz} (r,0)\,\,\,\,v(r,0) = \beta_{2} \tau_{z\varphi } (r,0) + r\Omega ,\,\,\,\,w(r,0) = 0,\, \\ & \quad - kT_{z} (r,0) = h_{f} (T_{w} - T(r,0)),\,\,\, - D_{B} C_{z} = h_{s} (C_{w} - C(r,0))\,\;\,\,\, - D_{n} N_{z} = h_{n} (N_{w} - N(r,0)), \\ & \quad u \to 0,\;\;v \to 0,\,\,\,T \to T_{\infty } ,\,\,\,\,C \to C_{\infty } ,\,\,N \to N_{\infty } . \\ \end{aligned}$$

Components of deformation rate tensors^2^ are:

11 $$\begin{aligned} {\text{e}}_{rr} & = 2u_{r} ,e_{\phi \phi } = 2\frac{u}{r},e_{zz} = 2w_{z} ,e_{r\phi } = e_{\phi r} = r\left( \frac{v}{r} \right)_{r} , \\ e_{z\phi } & = e_{\phi z} = v_{z} ,e_{rz} = e_{zr} = u_{z} + w_{r} , \\ \end{aligned}$$

and the components of the stress tensor is given by^7^:

12 $$\tau_{rr} = - p + \mu \left( {2u_{r} } \right) + \mu_{c} \left\{ {4\left( {u_{r} } \right)^{2} + \left( {u_{z} + w_{r} } \right)^{2} + \left( {v_{r} - \frac{v}{r}} \right)^{2} } \right\},$$

13 $$\tau_{zr} = \mu \left( {u_{z} + w_{r} } \right) + \mu_{c} \left\{ {\left( {2u_{r} } \right)\left( {u_{z} + w_{r} } \right) + v_{z} \left( {v_{r} - \frac{v}{r}} \right) + 2w_{z} \left( {u_{z} + w_{r} } \right)} \right\}$$

14 $$\tau_{\phi \phi } = - p + \mu \left( {2\frac{u}{r}} \right) + \mu_{c} \left\{ {\left( {4\frac{{u^{2} }}{{r^{2} }}} \right)\left( {v_{z} } \right)^{2} + \left( {v_{r} - \frac{v}{r}} \right)^{2} } \right\},$$

15 $$\tau_{r\phi } = \mu \left( {v_{r} - \frac{v}{r}} \right) + \mu_{c} \left\{ {\left( {2u_{r} } \right)\left( {v_{r} - \frac{v}{r}} \right) + \left( {2\frac{u}{r}} \right)\left( {v_{r} - \frac{v}{r}} \right) + 2v_{z} \left( {u_{z} + w_{r} } \right)} \right\},$$

16 $$\tau_{z\phi } = \mu \left( {v_{z} } \right) + \mu_{c} \left\{ {\left( {u_{z} + w_{r} } \right)\left( {v_{r} - \frac{v}{r}} \right) + 2\left( {\left( \frac{u}{r} \right)\left( {v_{z} } \right) + w_{z} \left( {v_{z} } \right)} \right)} \right\}.$$

Transformations are:

17 $$\begin{aligned} \eta & = \sqrt {\frac{\Omega }{\nu }} z,\,\,\,u = r\Omega f^{\prime}(\eta ),\,\,\,\,\,\,v = r\Omega g(\eta ),\,\,\,\,\,w = - 2\sqrt {\Omega \nu } f(\eta ), \\ \theta & = \frac{{T - T_{\infty } }}{{T_{w} - T_{\infty } }},\,\,\,\,\,\,\xi = \frac{{N - N_{\infty } }}{{N_{w} - N_{\infty } }},\,\,\,\,\,\,\,\,\varphi = \frac{{C - C_{\infty } }}{{C_{w} - C_{\infty } }}, \\ \end{aligned}$$

Using Eq. (17), Eqs. (3), (4), and (6)–(9) become:

18 $$f^{\prime\prime\prime} - f^{{\prime}{2}} + 2ff^{\prime\prime} + g^{2} - \lambda f^{\prime} + K(f^{{\prime\prime}{2}} - g^{{\prime}{2}} - 2f^{\prime}f^{\prime\prime}) = 0$$

19 $$g^{\prime\prime} - 2f^{\prime}g + 2fg^{\prime} - \lambda g - 2K(f^{\prime}g^{\prime\prime} - f^{\prime\prime}g^{\prime}) = 0,$$

20 $$\begin{aligned} & \theta^{\prime\prime} + \Pr f^{\prime}\theta + N_{b} \theta^{\prime}\phi^{\prime} + N_{t} \theta^{{\prime}{2}} \\ & \quad - \gamma \Pr (ff^{\prime\prime}\theta ^{\prime} + f^{2} \theta^{\prime\prime}) + Af^{\prime} + B\theta + N_{b} \theta^{\prime}\phi^{\prime} + N_{t} \theta^{{\prime}{2}} = 0, \\ \end{aligned}$$

21 $$\phi^{\prime\prime} + 2Scf\phi^{\prime} + \frac{{N_{b} }}{{N_{t} }}\theta^{\prime\prime} - K_{1} \phi (1 + \delta \theta )^{n} e^{{\frac{ - E}{{1 + \delta \theta }}}} = 0,$$

22 $$\xi^{\prime\prime} - Pe\left( {\xi^{\prime}\phi^{\prime} + \left( {\xi + \Omega_{1} } \right)\phi^{\prime\prime}} \right) + 2Lbf\xi^{\prime} = 0.$$

23 $$\begin{aligned} f^{\prime}(0) & = \alpha_{1} f^{\prime\prime}(0)\left[ {1 - 2Kf^{\prime}(0)} \right],\,\,\,\,\,f(0) = 0,\,\,\,\,\,\,g(0) = \alpha_{2} g^{\prime}(0)\left[ {1 - 2Kf^{\prime}(0)} \right] + 1,\,\;\;\theta^{\prime}(0) = - B_{1} (1 - \theta ),\, \\ \phi^{\prime} & = - B_{2} (1 - \phi ),\,\,\,\,\,\,\xi^{\prime} = - B_{3} (1 - \xi ), \\ f^{\prime}(\infty ) & = 0,\,\;\;g(\infty ) = 0,\theta (\infty ) = 0,\,\,\,\,\,\phi (\infty ) = 0,\,\,\,\,\xi (\infty ) = 0, \\ \end{aligned}$$

The quantities in the above equations are defined as:

24 $$\begin{aligned} \lambda & = \frac{\nu }{k\Omega }\,\gamma = \lambda_{1} \Omega ,\,\,\,\,N_{b} = \frac{{\tau D_{B} (C_{w} - C_{\infty } )}}{\nu },\,\,N_{t} = \frac{{\tau D_{T} \Delta T}}{{T_{\infty } \nu }},\,\,\,\,\,\,\,\alpha_{2} = \rho \sqrt {\Omega \nu } \beta_{2} ,\,\,\,\,B_{3} = \frac{{h_{n} }}{{D_{n} }}\sqrt {\frac{\nu }{\Omega }} ,\,\,\, \\ K & = \frac{{\mu_{c} \Omega }}{\mu },\,\,\,\,B_{1} = \frac{{h_{f} }}{k}\sqrt {\frac{\nu }{\Omega }} ,\,\,\,E = \frac{{E_{a} }}{{kT_{\infty } }},K_{1} = \frac{{k_{r}^{2} }}{\Omega },\,\,\,\alpha_{1} = \rho \sqrt {\Omega \nu } \beta_{1} ,\,\,\,\,\,\,B_{2} = \frac{{h_{s} }}{{D_{B} }}\sqrt {\frac{\nu }{\Omega }} ,\,\,\,\,\,\,\,\, \\ Pe & = \frac{{bW_{c} }}{{D_{m} }},\,\delta = \frac{\Delta T}{{T_{\infty } }},\,Lb = \frac{v}{{D_{m} }},\,\,\,\Omega = \frac{{N_{\infty } }}{{N_{f} - N_{\infty } }},\Pr = \frac{{\mu c_{p} }}{k},\,\,\,\,Sc = \frac{\nu }{{D_{B} }}. \\ \end{aligned}$$

Drag force coefficient, the rates of heat, mass, and the local motile microorganisms’ flux are defined by:

25 $$\begin{gathered} C_{f} = \frac{{\sqrt {\tau_{r}^{2} - \tau_{\varphi }^{2} } }}{{\rho u_{w}^{2} }},\,\,\,\,\,\,\,Sh_{x} = \frac{{rj_{w} }}{{D_{n} (C_{w} - C_{\infty } )}},\,\,\,\,\,\,Nu_{x} = \frac{{xq_{n} }}{{D_{m} (N_{w} - N_{\infty } )}}. \hfill \\ \hfill \\ \end{gathered}$$

The dimensionless Skin friction coefficient, rate of heat, and mass fluxes and are the local motile microorganisms’ flux are given as:

26 $$\begin{aligned} C_{f} \sqrt {{\text{Re}}_{x} } & = \sqrt {\left[ {f^{\prime\prime}(0))^{2} - (g^{\prime}(0)} \right]^{2} } ,\,\,\,\,\,\,\,\,\,Sh\sqrt {{\text{Re}}_{x} } = - \phi ^{\prime}(0). \\ Nu_{x} \sqrt {{\text{Re}}_{x} } & = - \xi ^{\prime}(0) \\ \end{aligned}$$

### Entropy generation analysis

Following the volumetric entropy generation is presented in^22^:

27 $$\begin{aligned} S_{G} & = \frac{k}{{T_{\infty }^{2} }}\left( {T_{z} } \right)^{2} + \frac{1}{{T_{\infty } }}\left( {\begin{array}{*{20}l} {2u_{r} \tau_{rr} + 2\left( {\frac{1}{r}v_{\phi } + \frac{u}{r}} \right)\tau_{\phi \phi } + 2w_{z} \tau_{zz} + 2\left( {\frac{1}{r}u_{\phi } + \left( \frac{v}{r} \right)_{r} } \right)\tau_{\phi r} } \hfill \\ { + w_{r} \tau_{rz} + \frac{1}{r}w_{\phi } \tau_{z\phi } } \hfill \\ \end{array} } \right) \\ & \quad + \frac{{R_{D} }}{{T_{\infty } }}T_{z} C_{z} + \frac{{R_{D} }}{{C_{\infty } }}(C_{z} )^{2} + \frac{{R_{D} }}{{n_{\infty } }}\left( {N_{z} } \right)^{2} + \frac{{R_{D} }}{{T_{\infty } }}N_{z} C_{z} + \frac{{\mu u^{2} }}{{T_{\infty } k}}. \\ \end{aligned}$$

The entropy generation in the dimensionless form can be derived as:

28 $$\begin{aligned} N_{G} & = \frac{{S_{G} }}{{S_{0}^{\prime \prime \prime } }} = \Omega_{1} \theta^{\prime 2} + Br\left( {\left[ {\frac{1}{{\text{Re}}}f^{\prime 2} + \lambda f^{\prime 2} + \frac{16}{{\text{Re}}}Kf^{\prime 3} - 2Kf^{\prime 3} - 2Kf^{\prime } f^{\prime \prime 2} - 2Kf^{\prime } g^{\prime 2} } \right]} \right) \\ & \quad + L\theta^{\prime } \phi^{\prime } + L\frac{{\Omega_{2} }}{{\Omega_{1} }}\phi^{\prime 2} + L_{1} \xi^{\prime } \theta^{\prime } + L_{1} \frac{{\Omega_{3} }}{{\Omega_{1} }}\xi^{\prime 2} . \\ \end{aligned}$$

where

29 $$\begin{aligned} Br & = \frac{{\mu \Omega^{2} }}{{k(T_{0} - T_{\infty } )}},\,\,\,\,\,\,\,\sigma_{1} = \frac{{(T_{0} - T_{\infty } )}}{{T_{\infty } }},\,\,\,\,\,L = \frac{{R_{D} \left( {C_{w} - C_{\infty } } \right)}}{k},\,\,\,\,\;L_{1} = \frac{{R_{D} (N_{w} - N_{\infty } )}}{k}, \\ \sigma_{2} & = \frac{{(C_{w} - C_{\infty } )}}{{C_{\infty } }},\,\,\,\,\sigma_{3} = \frac{{(N_{w} - N_{\infty } )}}{{N_{\infty } }},\,\;\;S^{\prime \prime \prime }_{0} = \frac{{k(T_{0} - T_{\infty } )}}{{\nu T_{\infty } }}. \\ \end{aligned}$$

## Graphical and tabulated outcomes with discussion

This sector (Figs. 2, 3, 4, 5, 6, 7, 8, 9, 10, 11, 12, 13, 14, 15, 16, 17, 18, 19, 20, 21) is dedicated to examining the influence of varied evolving parameters on the flow model involved profiles. The ranges of the parameters are defined as:

$$\begin{aligned} & 0.0 \le K \le 0.7,0.5 \le \alpha_{1} ,\alpha_{2} \le 2.0,0.1 \le \lambda \le 0.7,0.4 \le B_{1} \le 0.7,0.5 \le \gamma \le 2.0, - 0.9 \le A \le 0.9, - 0.4 \le B \le 0.4, \\ & 0.1 \le \delta \le 1.3,0.0 \le n \le 3.0,0.1 \le E \le 0.7,0.4 \le Lb \le 0.7,1.0 \le Br \le 5.0,0.5 \le \Omega_{2} \le 0.8,0.5 \le {\text{Re}} \le 3.0. \\ \end{aligned}$$

**Figure 2:**
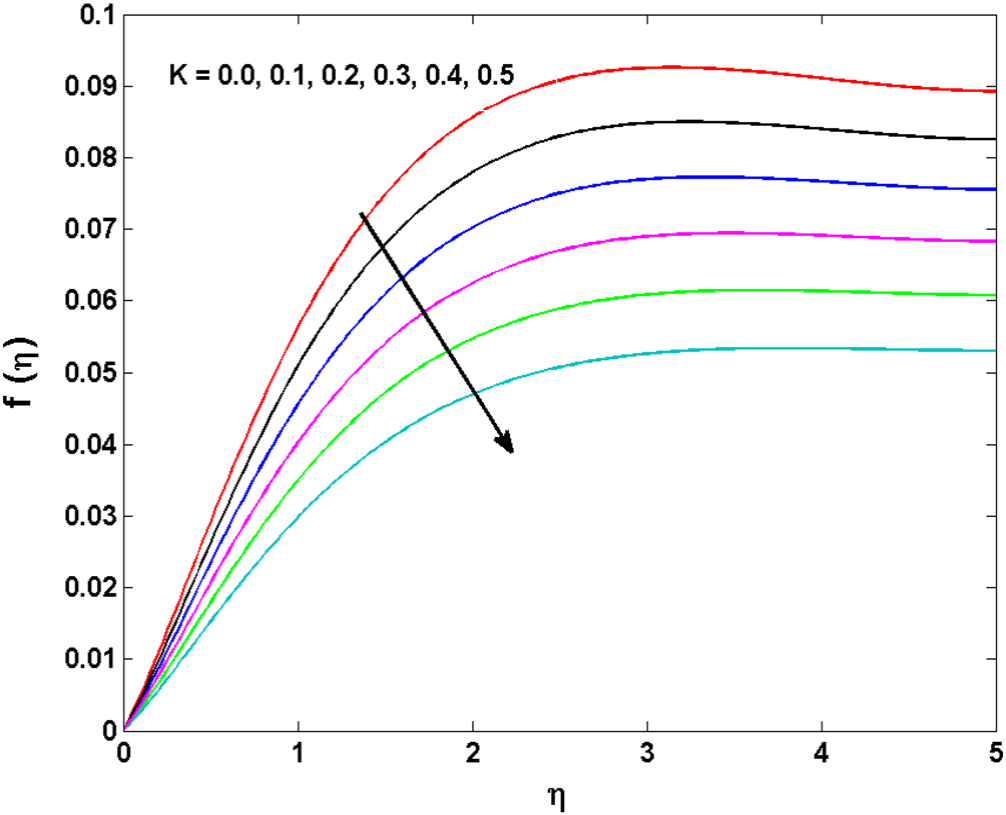
Variations of $K$ to $f(\eta )$.

**Figure 3:**
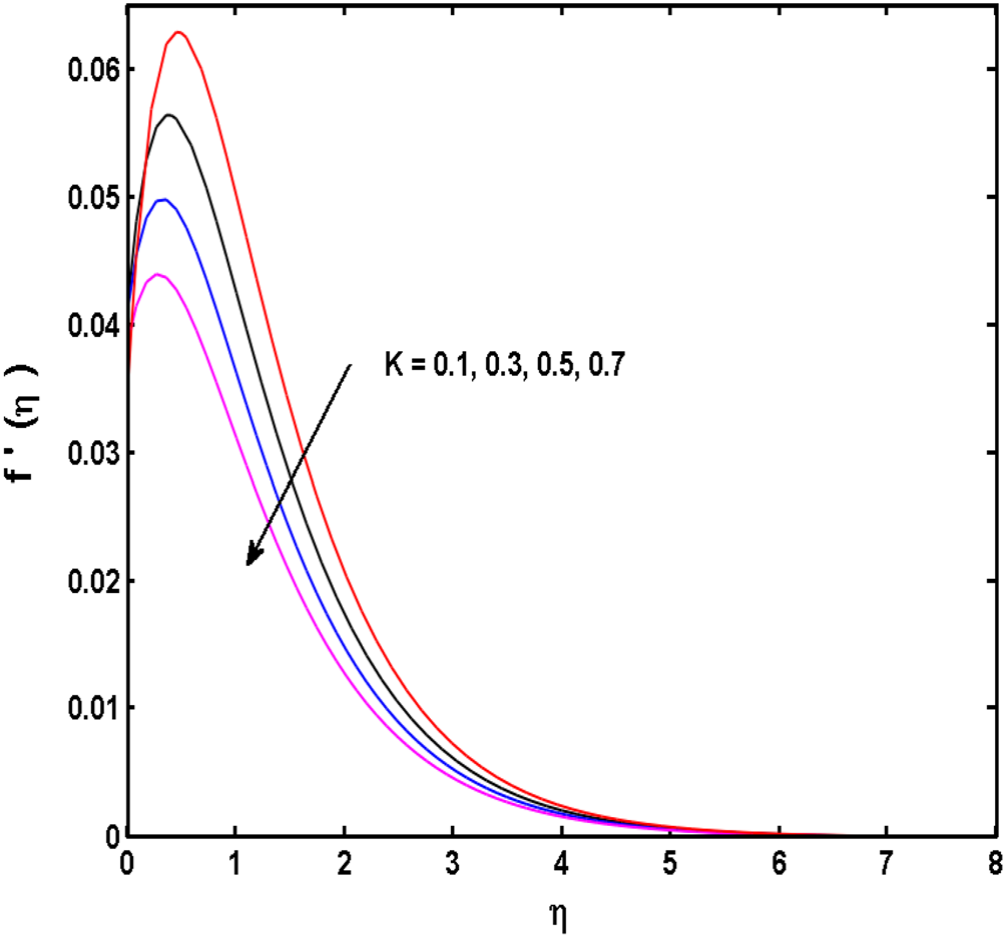
Variations of $K$ to $f^{\prime}\,(\eta )$.

**Figure 4:**
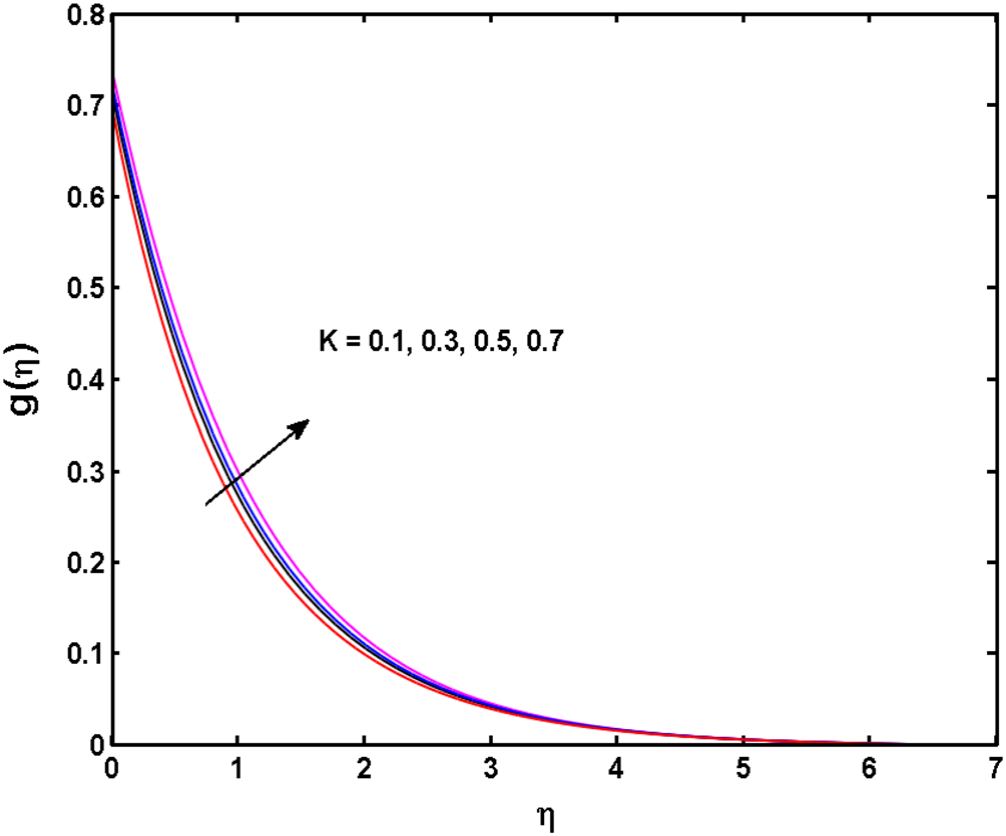
Variations of $K$ to $g\,(\eta )$.

**Figure 5:**
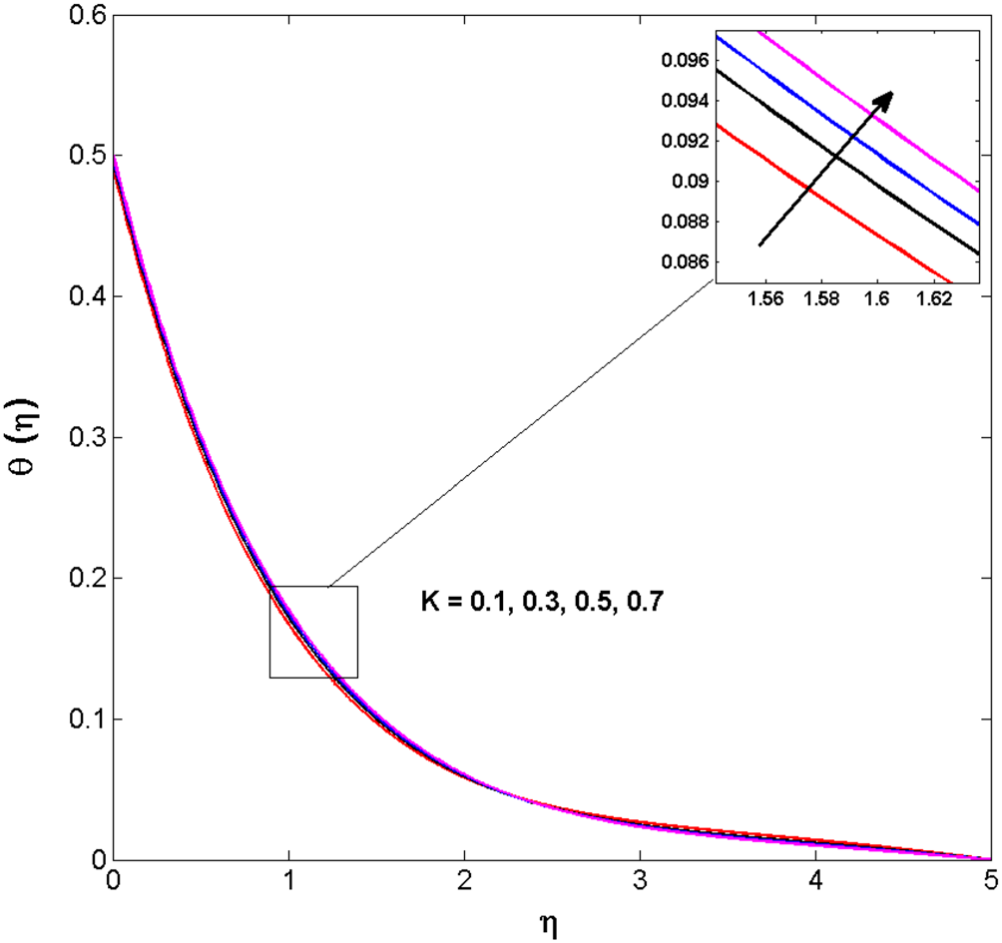
Variations of $K$ to $\theta \,(\eta )$.

**Figure 6:**
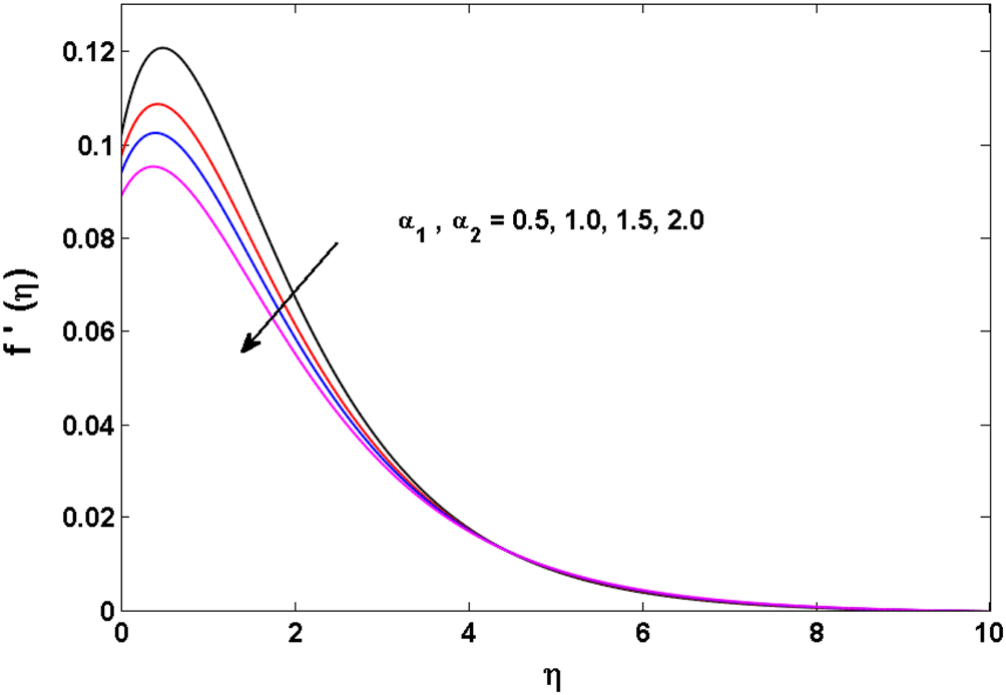
Variations of $\alpha_{1} ,\,\,\alpha_{2}$ to $f^{\prime}\,(\eta )$.

**Figure 7:**
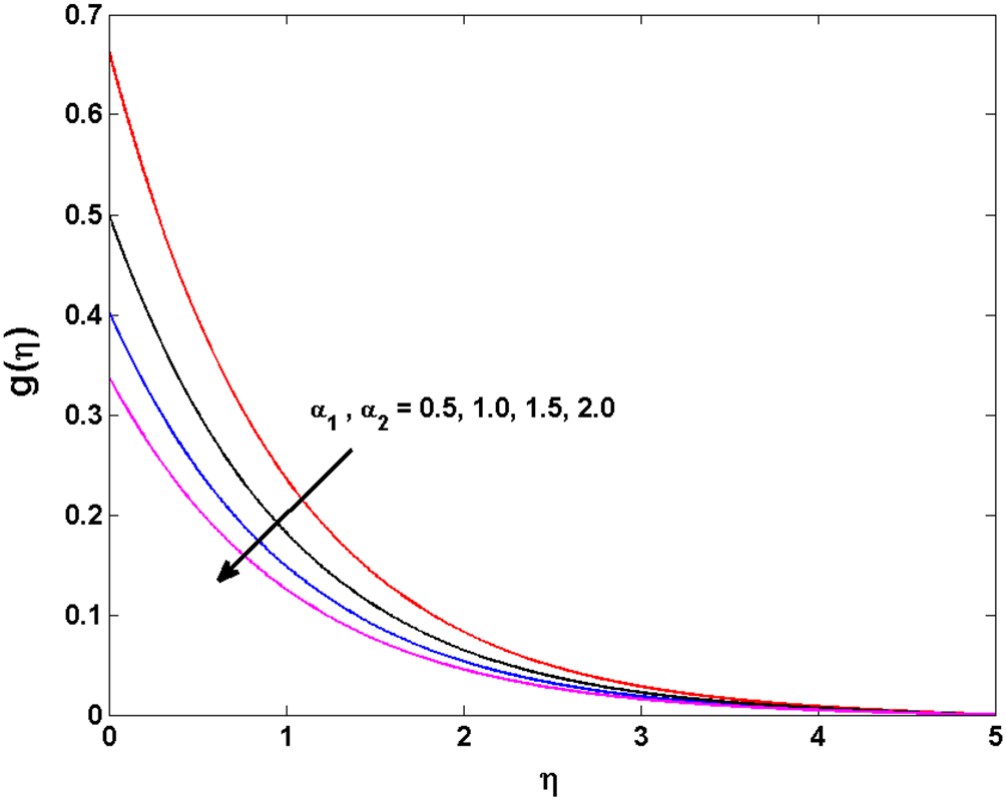
Variations of $\alpha_{1} ,\,\,\alpha_{2}$ to $g\,(\eta )$.

**Figure 8:**
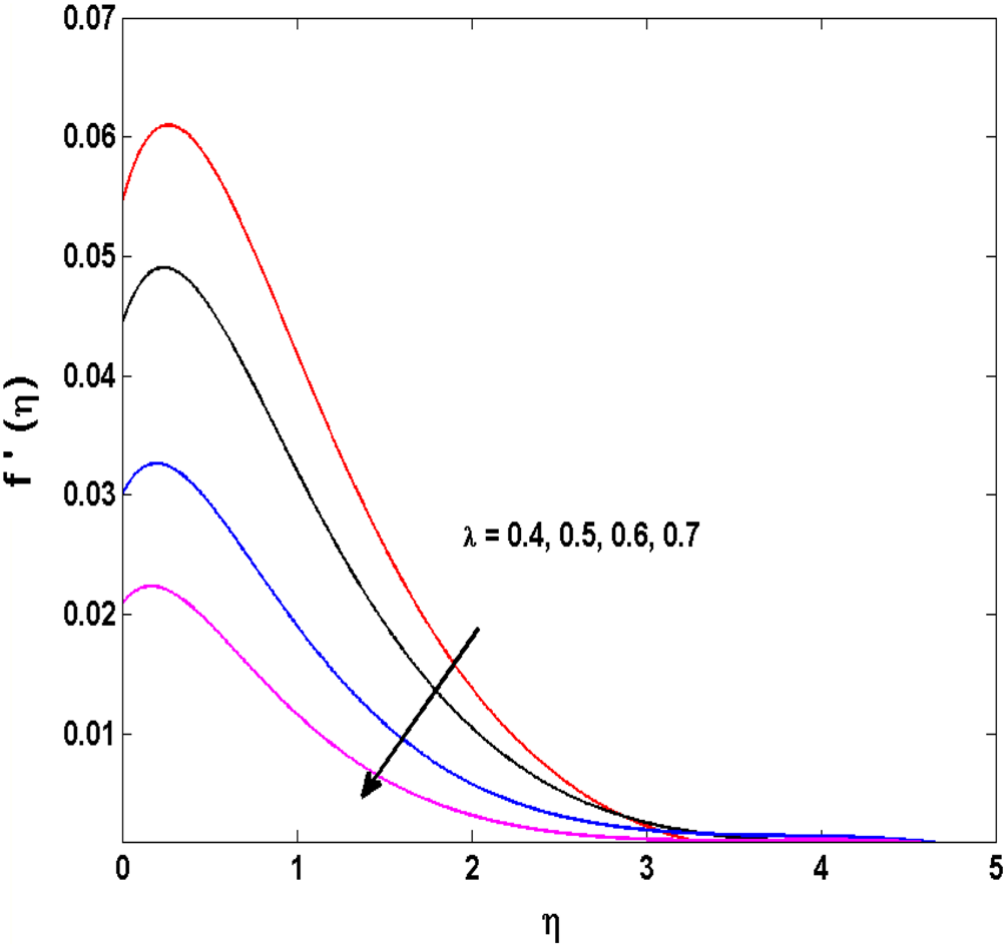
Variations of $\lambda$ to $f^{\prime}\,(\eta )$.

**Figure 9:**
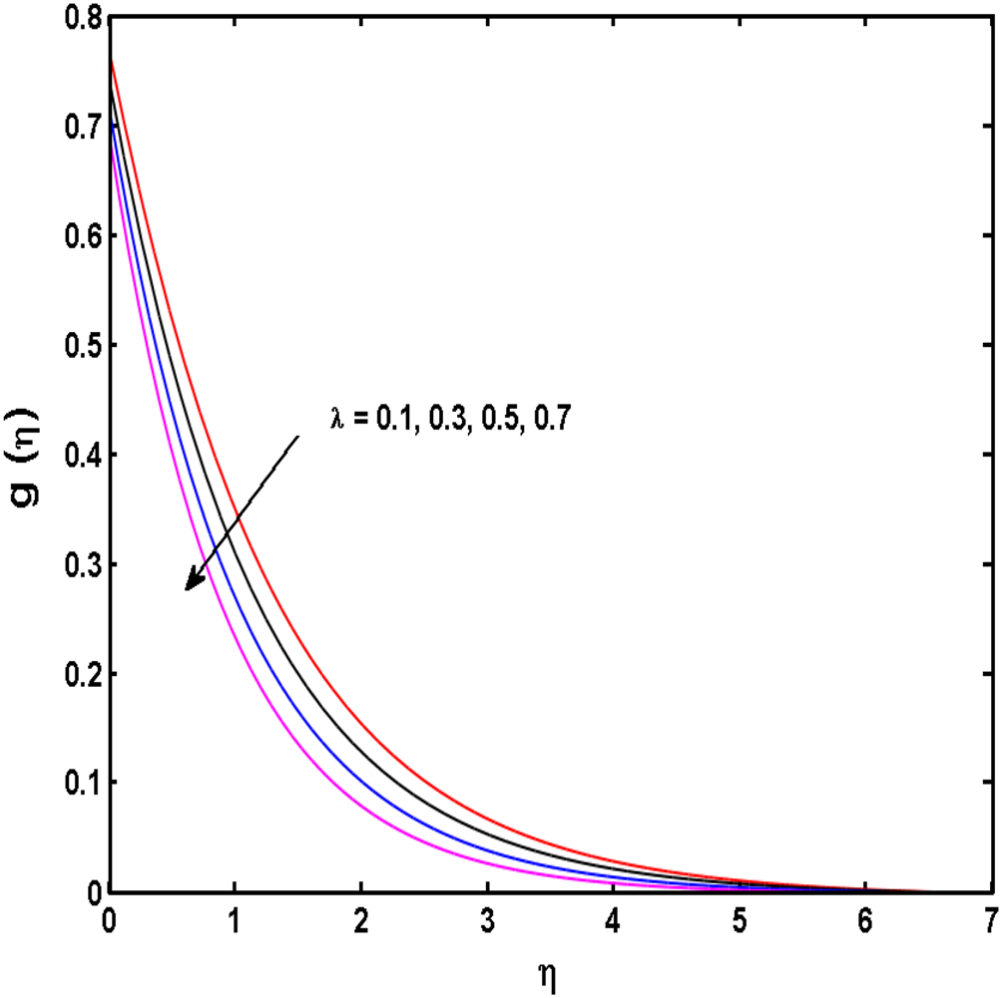
Variations of $\lambda$ to $g\,(\eta )$.

**Figure 10:**
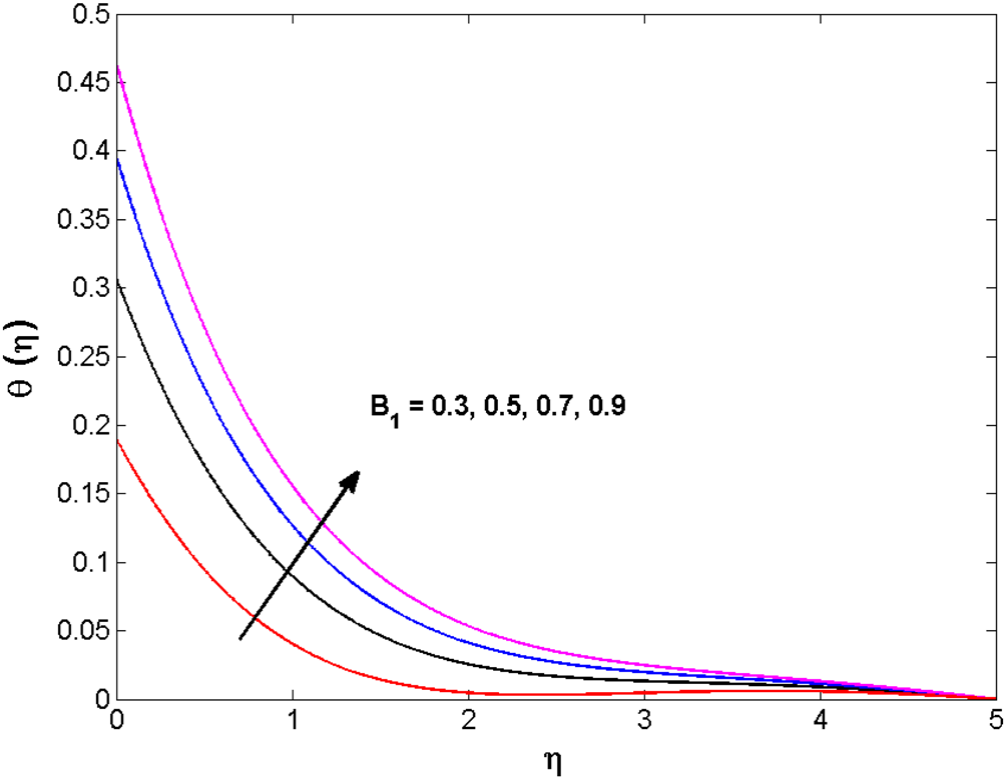
Variations of $B_{1}$ to $\theta \,(\eta )$.

**Figure 11:**
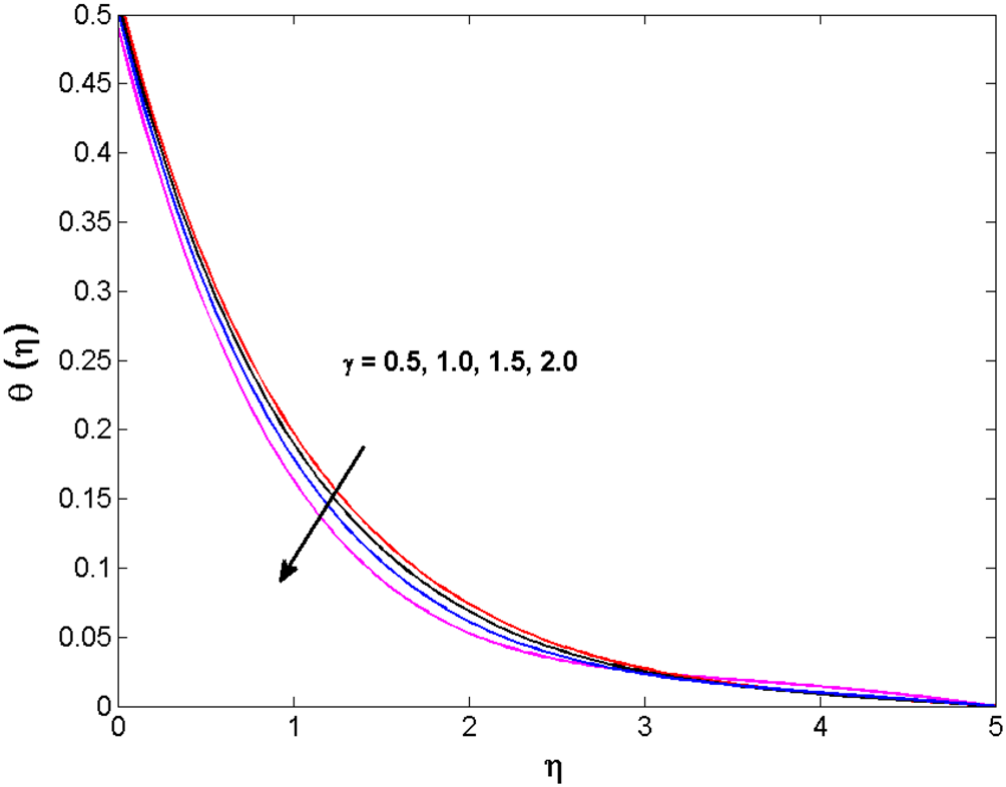
Variations of $\gamma$ to $\theta \,(\eta )$.

**Figure 12:**
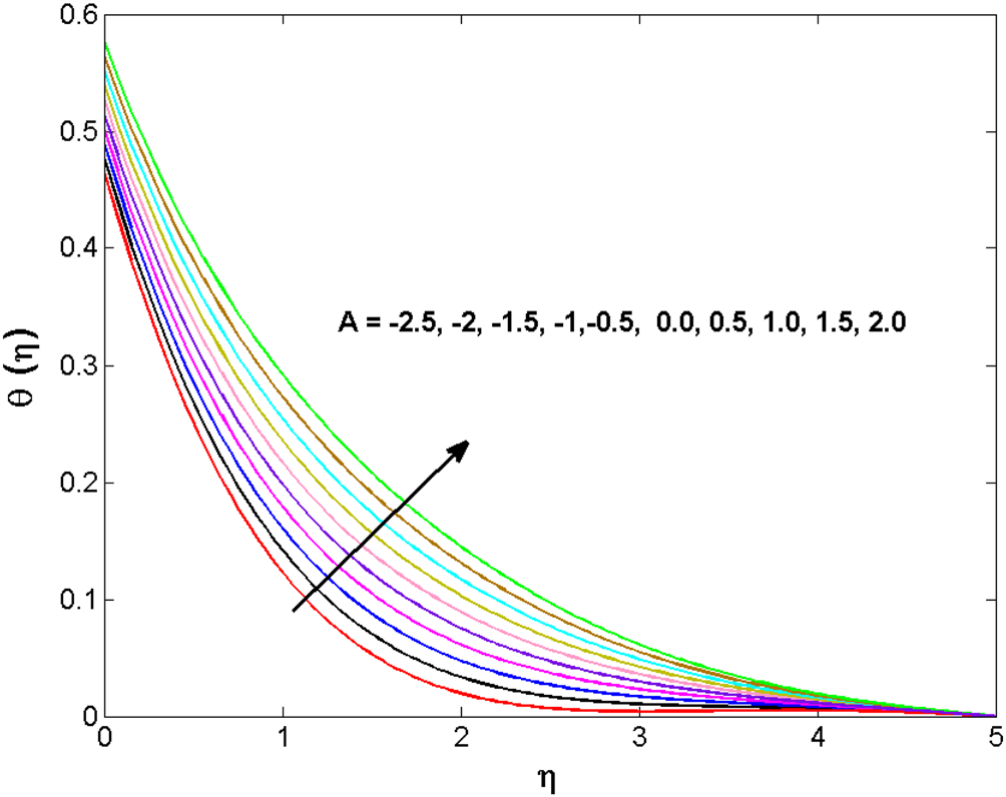
Variations of $A$ to $\theta \,(\eta )$.

**Figure 13:**
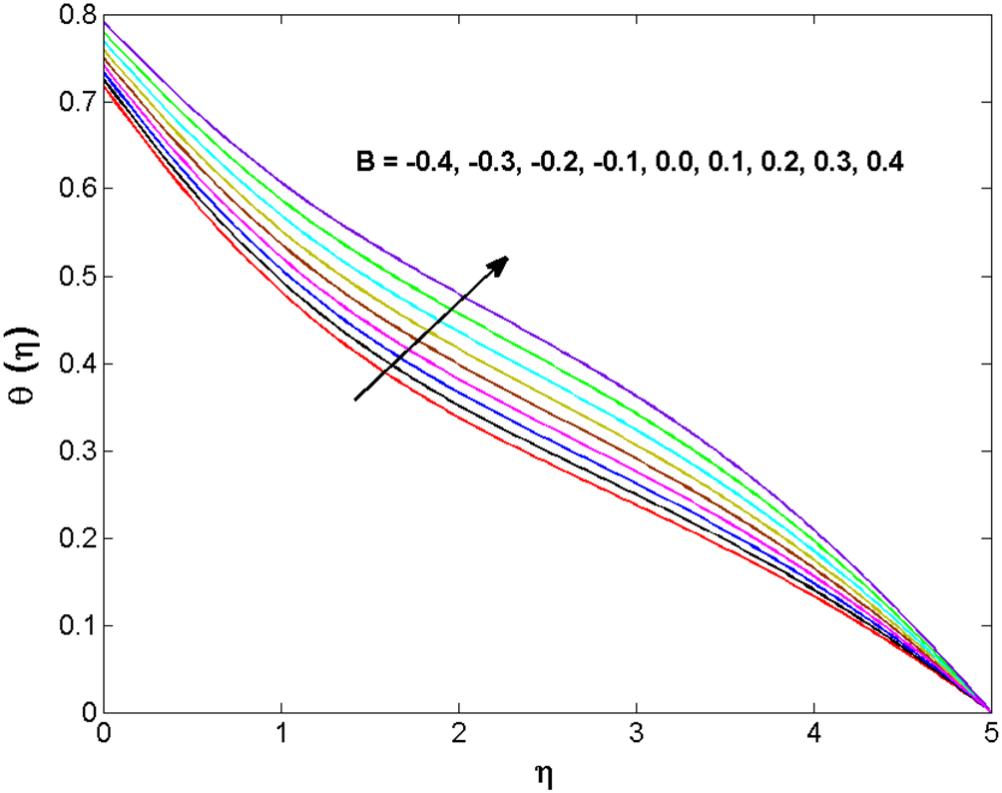
Variations of $B$ to $\theta \,(\eta )$.

**Figure 14:**
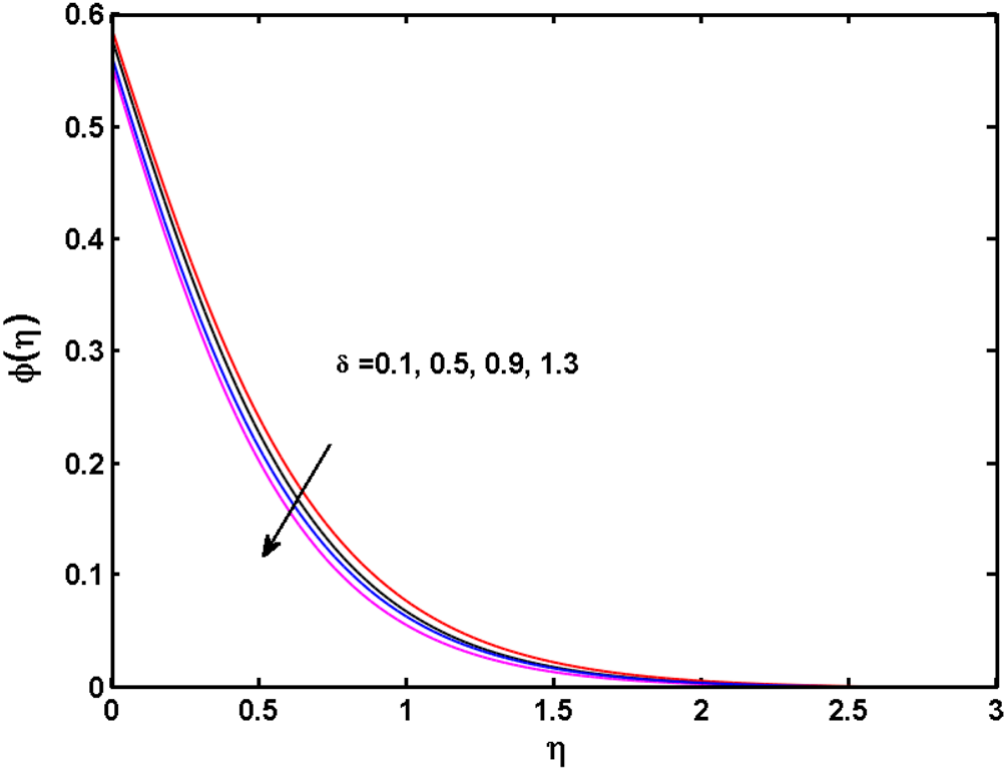
Variations of $\delta$ to $\phi \,(\eta )$.

**Figure 15:**
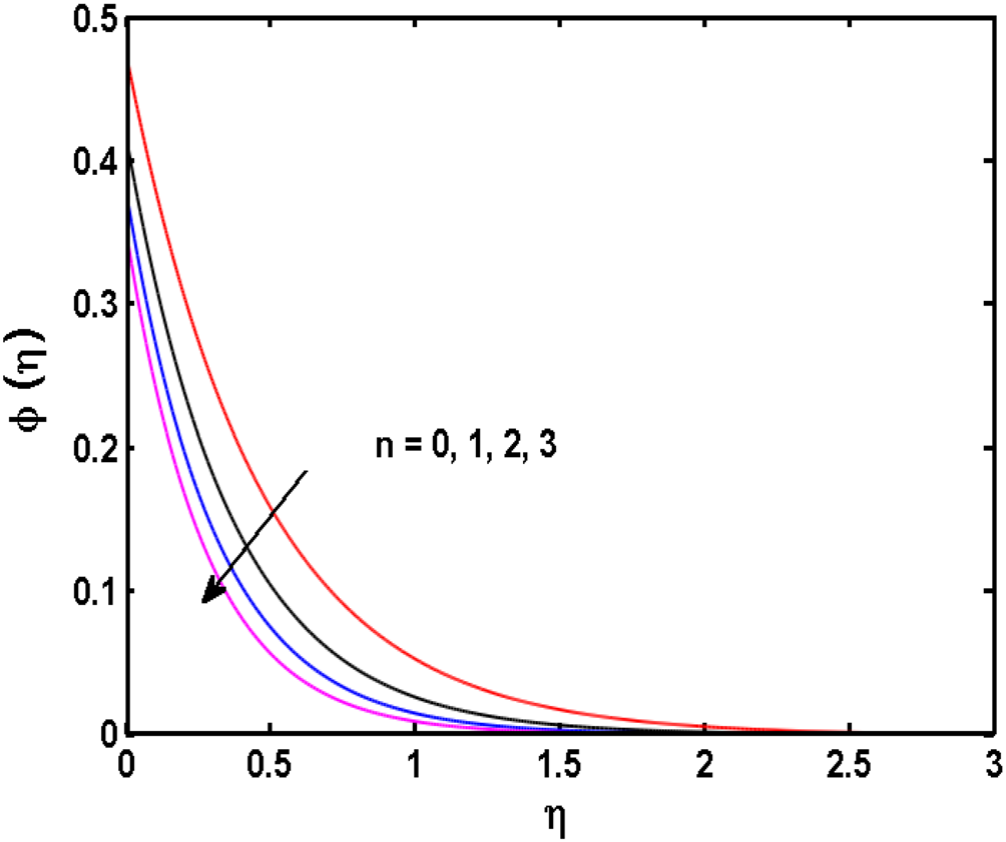
Variations of $n$ to $\phi \,(\eta )$.

**Figure 16:**
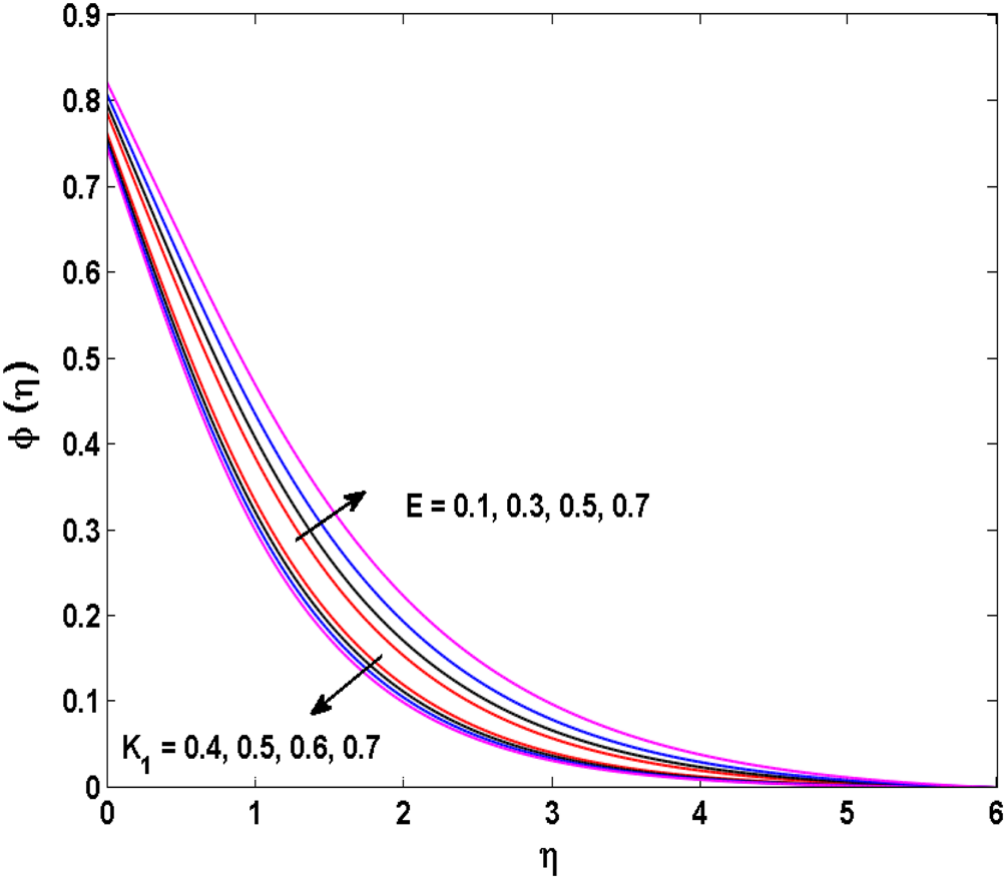
Variations of $K_{1}$ and $E$ to $\phi \,(\eta )$.

**Figure 17:**
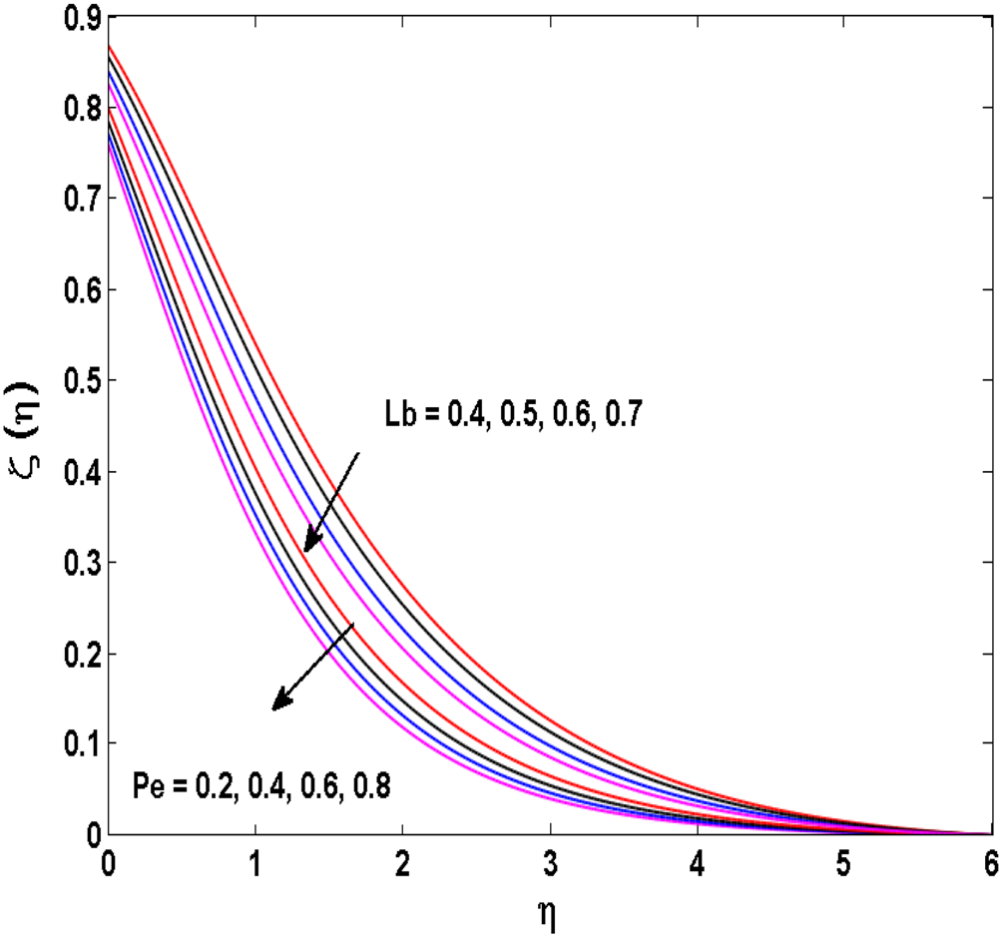
Variations of $Pe$ and $Lb$ to $\zeta \,(\eta )$.

**Figure 18:**
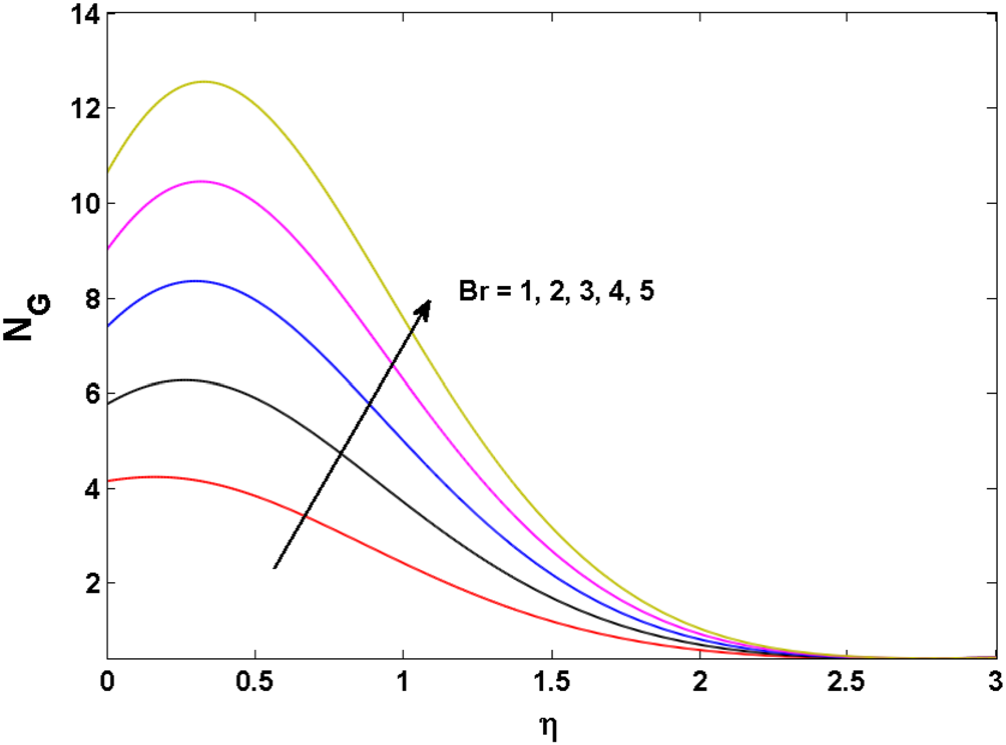
Variations of $Br$ to $N_{G}$.

**Figure 19:**
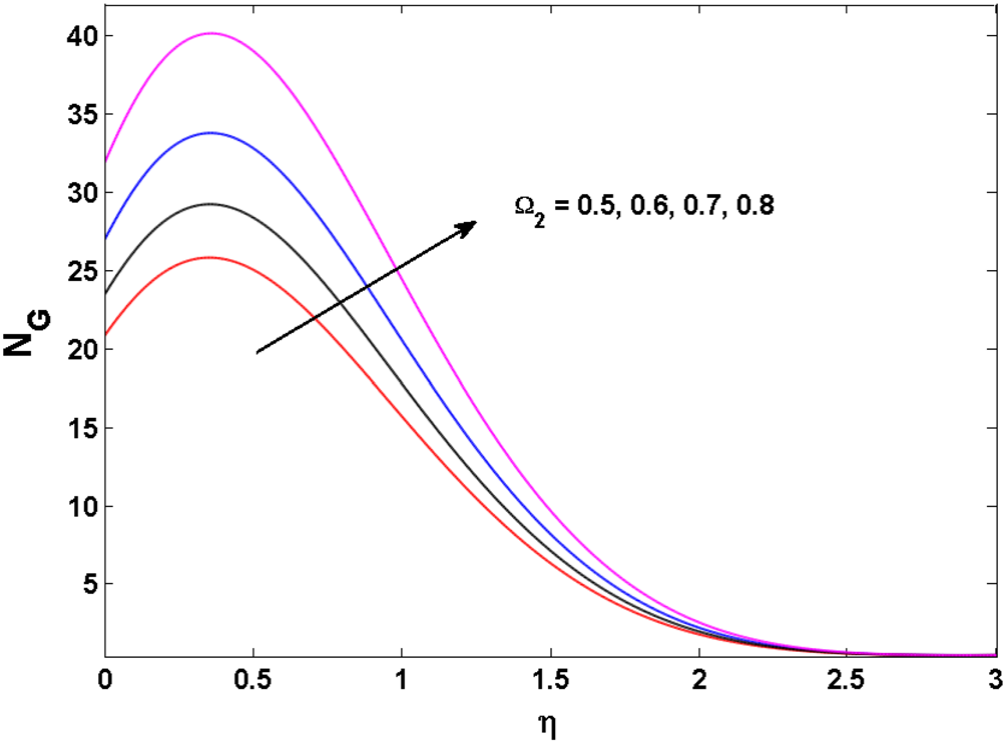
Variations of $\Omega_{2}$ to $N_{G}$.

**Figure 20:**
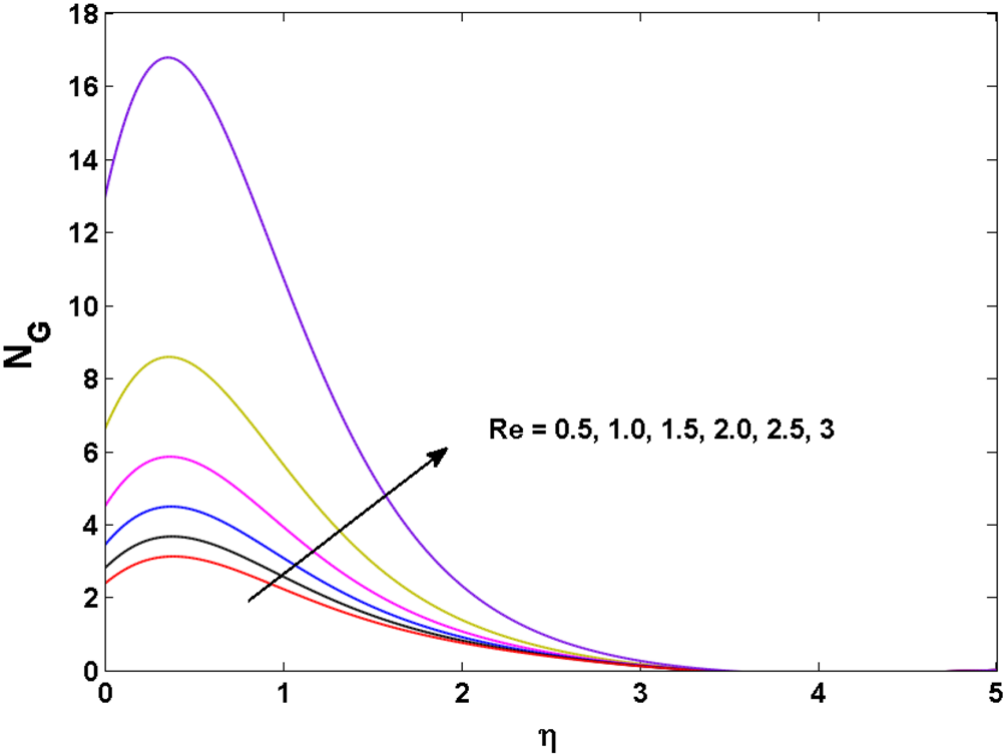
Variations of ${\text{Re}}$ to $N_{G}$.

**Figure 21:**
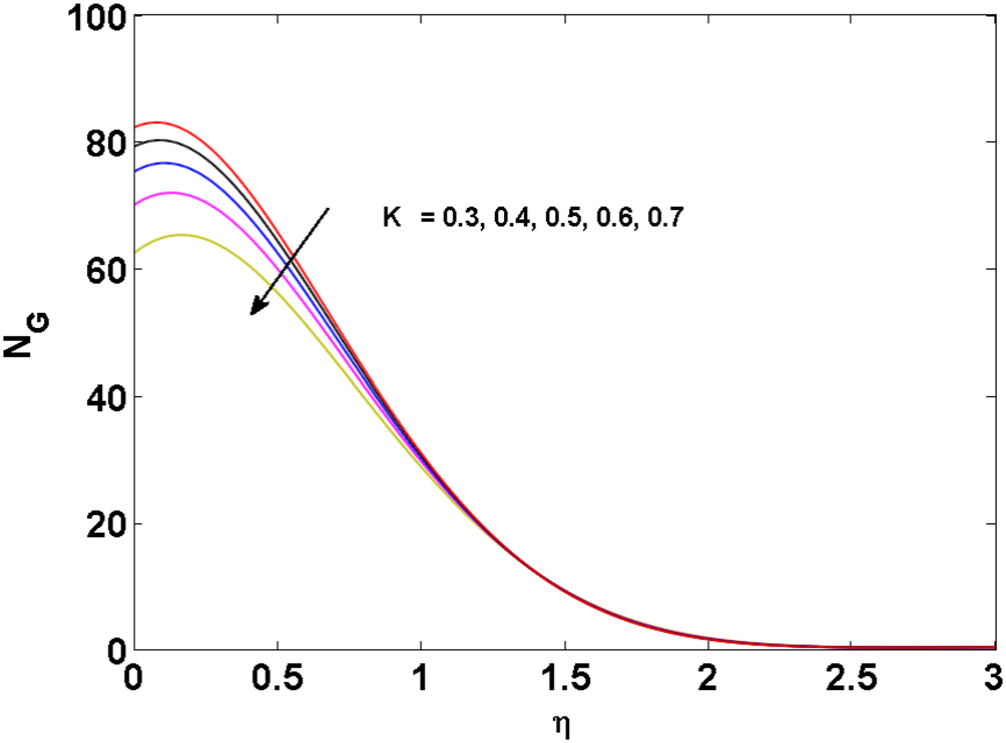
Variations of $K$ to $N_{G}$.

The behavior of Reiner–Rivlin fluid parameter K on the axial, radial, and tangential velocities, and temperature profile is revealed in Figs. 2, 3, 4 and 5 for the uniform wall roughness. It is noticed from Figs. 2 and 3 that the axial and radial velocities are dwindled near the disk and augmented when striding far afield from it. As the fluid move in the direction of the axis of rotation, the centrifugal forces along with viscoelastic effects generates a wake flow in the radial direction and a lesser amount of liquid is seen along an axial path. That is why the axial velocity profile attains its maximum near the disk and diminishes away from the disk. However, contrary to axial and radial profiles, the azimuthal velocity profile attains an opposite trend (Fig. 4). Similarly, in Fig. 5 the fluid temperature is enhanced for growing estimates of K. Here, the thermal boundary layer thickness expands when the comparatively cold water is taken along the axial direction. Figures 6 and 7 represent the outcomes of the wall slip parameters $\alpha_{1} ,\alpha_{2}$ versus the radial and tangential velocity components when K = 1. Here, it is further assumed that estimates of both $\alpha_{1} ,\alpha_{2}$ are equal. It is noticed that the radial velocity is more away from the disk for large values of $\alpha_{1} ,\alpha_{2}$ and an opposing trend is seen near it. The azimuthal velocity component possesses a reverse impact for the estimates of $\alpha_{1} ,\alpha_{2} .$ This is because of the disk’s rotational impact that is partly transferred to the adjacent boundary layer. The impact of the permeability parameter $\lambda$ versus radial and azimuthal distributions is shown in Figs. 8 and 9. It is noticed that both velocity profiles dwindle for large values of $\lambda$. Physically, the movement of the liquid is hindered due to the occurrence of permeable media, and as a result, a falloff in the fluid velocities is seen.

The behavior of the conjugate parameter $B_{1}$ on temperature distribution is studied in Fig. 10. It is noticed that temperature and thermal boundary layer thickness are boosted for large estimates of $B_{1}$. Here, the increment in $B_{1}$ instigating the heat transfer that pushes extra heat from the surface. In this way, the temperature is enhanced. Figure 11 is illustrated to show the influence of the thermal relaxation parameter $\gamma$ on the temperature profile. It is noted that the fluid temperature is decreased for large values of the $\gamma .$ Large values of the $\gamma$ points out the strong characteristics of the insulating material which are responsible for the drop in the fluid temperature. The influence of $A$ and $B$ (heat source and sink) on the temperature distribution is analyzed in Figs. 12 and 13. It is perceived that the temperature profile is rising when the values of both $A,B$ are increased. Since the occurrence of heat source parameter produces more heat within the nanofluid flow field which leads to an increment in the boundary layer thickness. Hence, more heat in the nanofluid enhances the fluid temperature. Figures 14 and 15 are drawn to witness the impact of temperature difference parameters $\delta$ and $n$ on the concentration distribution profile. It is witnessed that concentration distribution is a diminishing function for both $\delta$ and $n$. This shows that the concentration boundary layer thickness growing when the difference between ambient and wall temperatures is increased. The influence of the chemical reaction parameter $K_{1}$ and the activation energy parameter $E$ against the concentration profile is portrayed in Fig. 16. It is seen that the concentration of the fluid weakens once the estimates of $K_{1}$ are augmented. Large values of $K_{1}$ are associated with the destructive chemical reaction and this eventually dissolves the fluid species. That is why the liquid concentration is reduced and an opposite behavior is noticed for $E.$ Here, the generative case is strengthened for large estimates of $E.$ As a result concentration is enhanced. The variation of bioconvection Lewis number $Lb,$ and Peclet number $Pe,\,$ on the fluid motile density is revealed in Fig. 17. It is detected that the motile density profile decreases for large values of the Peclet number. For the higher $Pe,$ fluid motile density is decreased due to the decline in the diffusivity of microorganisms. It is also observed for mounting estimations of $Lb,$ causes falloffs of microorganisms hence the profile of motile density also drops in this case.

Figures 18, 19, 20 and 21 display the vital role of volumetric entropy generation for varied estimations of $Br,\Omega_{2} ,{\text{Re}}$ and $K$. For greater $Br,\Omega_{2}$ and ${\text{Re}}$ entropy generation is increases. For large values of $Br$ viscous dissipation generates less transfer rate and thus augments entropy generation rises. For $Br = 0,$ viscous dissipative irreversibility disappears and only heat transfer irreversibility produce. The effect of Re on entropy generation is expressed in Fig. 20. For large estimates of the Reynolds number, the substantial motion of the fluid molecules is witnessed. Thus, escalating the entropy generation rate. Figure 21 reveals that for higher values of the Reiner–Rivlin parameter $K$ entropy generation is declined.

Table 2 represents the numerical outcomes for skin friction coefficient, azimuthal velocity, entrainment velocity, and radial velocity for varied estimates of the slip and the Reiner–Rivlin parameters. Here, $f(\infty )$ helps in finding the volumetric flow rate of the von-Karman problem. The driving torque or the disk’s torque is gauged through $g^{\prime}(0).$ The viscoelastic impacts affect the torque in the Von Karman flow. The surface drag force coefficient and the resisting torque are enhanced for growing values of the radial and the tangential velocity components. It is comprehended that high torque at the disk shaft is enhanced for elevated values of the tangential slip parameter. It is also inferred from the tabulated values that skin friction coefficient and driving torque possess an increasing tendency for the Reiner–Rivlin parameter. However, an opposing trend is seen in the case of the volumetric flow rate. Table 3 portrays different variations of the rate of mass flux for different values of $K,N_{t} ,N_{b} ,\Pr$ and *Sc*. It is seen that for the large values of $K,N_{b}$ and $\Pr$ Sherwood number decreases and the opposing effects are seen for $N_{t}$ and $Sc.$ Table 4 portrays different estimations in local density profile for various values of $B_{1}$, $K,Pe$ and $Lb$. It is observed that for large estimates of $B_{1}$, $Lb$ and *Pe* the local density number of microorganisms increases while falls off for greater parameter. Table 5 computes the numerical values of $f(\infty )$ for the increasing wall roughness parameter and Reiner–Rivlin parameter. The results obtained by using bvp4c are compared with Naqvi et al.^8^. An excellent harmony among both results is achieved.

**Table 2:** The tabulation of numerical results for Drag force $C_{F} {\text{Re}}_{x}^{1/2}$ against the various values of $\alpha_{1} ,\alpha_{2} ,K,$ parameters.

$\alpha_{1}$	$\alpha_{2}$	$K$	$\sqrt {(f^{\prime\prime}(0))^{2} + (g^{\prime}(0))^{2} }$	$g^{\prime}(0)$	$f(\infty )$	$f^{\prime\prime}(0)$
1	2	0.2	0.3101282	− 0.30858225	0.058277735	0.030927356
1.5	2		0.3111269	− 0.31012459	0.063644215	0.024953560
2	2		0.3749075	− 0.31115555	0.067213467	0.020913603
2	2		0.3749075	− 0.31115555	0.067213467	0.020913603
2	3		0.2353182	− 0.23494064	0.044550026	0.013326333
2	4		0.1895022	− 0.18927773	0.031514276	0.009220524
2	2	0.3	0.3126156	− 0.31199346	0.063018309	0.019713295
		0.7	0.3131332	− 0.31279718	0.045216515	0.014503586
		1	0.3139463	− 0.31364883	0.031017367	0.010366372

**Table 3:** Numerical results for $Sh_{x} {\text{Re}}^{ - 0.5}$ against the various values of the $K$, $N_{t}$, $N_{b}$, $\Pr$, $Sc$, and $\beta$ parameters.

$K$	$N_{t}$	$N_{b}$	$\Pr$	$Sc$	$- \varphi ^{\prime}(0)$
0.2	0.1	0.6	3	4	0.37790968
0.7					0.36797258
1					0.36235873
	0.3				0.42296784
	0.4				0.44798048
	0.5				0.47456857
		0.15			0.42183083
		0.2			0.40723295
		0.3			0.39261496
			4		0.3945757
			6		0.38030306
			8		0.36270411
				1	0.3945757
				3	0.49663337
				5	0.55345741
					0.39615661
					0.3945757
					0.39407366

**Table 4:** The tabulation of numerical results for $Nu_{x} {\text{Re}}^{ - 0.5}$ against the various values of the $K$, $Pe,$ and $Lb$ parameters.

$B_{1}$	$K$	$Pe$	$Lb$	$- \xi ^{\prime}(0)$
0.2	0.2	0.2	1	0.17235092
0.3				0.23692559
0.4				0.29148902
	0.2			0.42313856
	0.7			0.39628911
	1			0.37841022
		0.3		0.34334759
		0.4		0.36772149
		0.5		0.39067931
			1.5	0.32953031
			2	0.34081314
			2.5	0.35143172

**Table 5:** The tabulation of numerical results of $f(\infty )$ by keeping $\Pr = 7,Sc = 0.5,$ $N_{t} = 0.1,N_{b} = 0.6$.

$\alpha_{1}$	$\alpha_{2}$	$K$	Naqvi et al.^8^ $f(\infty )$	Present results bvp4c $f(\infty )$
1	2	1	0.289891	0.289893
1.5	2	1	0.298954	0.298958
2	2	1	0.305295	0.305298
2	2	1	0.305295	0.305299
2	3	1	0.277820	0.277860
2	4	1	0.256126	0.256130
2	2	0.2	0.372886	0.372889
2	2	0.7	0.317290	0.317299
2	2	0.9	0.052950	0.053000

## Final remarks

In this study, the flow of the Reiner–Rivlin nanofluid generated by a rotating disk in the presence C–C heat flux and the gyrotactic microorganisms is deliberated. The flow is also accompanied by the effects of the heat source/sink, chemical reaction with activation energy with slip, and convective boundary conditions. The problem is solved numerically. The noteworthy observations of the model are appended as below:

- The radial and axial velocity components are diminishing functions for the Reiner–Rivlin parameter. However, an adverse impact is witnessed in the case of azimuthal velocity, and temperature profile.
- A diminishing impact of the slip parameters is perceived for both the radial and azimuthal velocity components.
- The permeability parameter shows dwindling influence versus the radial and tangential velocity components.
- Higher estimations of the thermal relaxation parameter cause reduction in the temperature profile.
- The fluid temperature is escalated for mounting estimates of the non-uniform heat source/sink parameter.
- The chemical reaction and the activation energy parameters possess an opposing impact on the concentration profile.
- The motile density profile diminishes for large values of the Peclet and the bioconvective Lewis numbers.
- The Brinkman and Reynolds numbers show opposing trends versus volumetric entropy generation.

## List of symbols

$$C_{f}$$: Drag force
$B_{2}$: Convection diffusion parameter
$$(u,v,w)$$: Velocity components
$E_{a}$: Activation energy coefficient
$K_{r}$: Chemical reaction rate constant
$c_{p}$: Capacity of specific heat
$$Sc$$: Schmidt number
$$K$$: Fluid parameter
$D_{B}$: Coefficient of Brownian diffusion
$$Sh_{x}$$: Sherwood number
$T_{w}$: Temperature on wall
$$Br$$: Brinkman number
$Wc$: Constant speed of cell swimming
$C_{\infty }$: Concentration in the free stream
$u_{w}$: Axial route having a velocity
$$L$$: Diffusion parameter
*Re*: Reynolds number
$Nu_{x}$: Local density number
$q^{*}$: Non uniform heat source/sink
$C_{s}$: Solid surface heat capacity
*Pe*: Peclet number
$$\kappa$$: Thermal conductivity
$$T$$: Fluid temperature
$N_{b}$: Brownian motion parameter
$$N_{G}$$: Entropy rate
*D*_*T*_: Coefficient of thermophoretic diffusion
$E$: Activation energy parameter
*T*_*∞*_: Diffusive temperature
$h_{f}$: Convective heat transfer coefficient
$h_{s}$: Mass transfer coefficient
$$\Pr$$: Prandtl number
$$N_{t}$$: Thermophoresis parameter
$A,B$: Space and temperature-dependent heat generation and absorption parameters
$$D_{m}$$: Microorganism’s diffusivity
$$C$$: Fluid concentration
$$Lb$$: Lewis number
$$B_{1}$$: Biot number
$$Sc$$: Schmidt number
$$L_{{}} ,L_{1}$$: Diffusion parameters
$A^{*} ,B^{*}$: Source, sink coefficients
$$Br$$: Brinkman number
$N_{\infty }$: Ambient motile density

## Greek letters

$\gamma$: Thermal relaxation time
$$\Omega$$: Angular velocity
$\rho$: Density of fluid
$\sigma_{1} ,\sigma_{2} ,\sigma_{3}$: Temperature, concentration and motile ratio parameter
$$\nu$$: Kinematic viscosity
$$\lambda$$: Permeability parameter
$\mu$: Liquid dynamic viscosity
$\beta_{1} ,\beta_{2}$: Radial and azimuthal slip parameters
$\alpha_{1} ,\alpha_{2}$: Radial and azimuthal slip coefficients
$\lambda_{2}$: Thermal relaxation factor
$\delta$: Ratio of the diffusion coefficient
$$\tau_{w}$$: Shear stress ($${\text{kg}}/{\text{ms}}^{2}$$)
